# The use of automated insulin delivery system in people with type 1 diabetes and end stage kidney disease on hemodialysis

**DOI:** 10.1210/jcemcr/luag151

**Published:** 2026-06-01

**Authors:** Ramita Anakepeerasak, Jutipond Jitchana, Nitchakarn Laichuthai

**Affiliations:** Department of Medicine, Faculty of Medicine, Chulalongkorn University, Bangkok 10330, Thailand; Division of Endocrinology and Metabolism, Department of Medicine, Faculty of Medicine, Chulalongkorn University, Bangkok 10330, Thailand; Division of Endocrinology and Metabolism, Department of Medicine, Faculty of Medicine, Chulalongkorn University, Bangkok 10330, Thailand; Division of Endocrinology and Metabolism, Department of Medicine, Faculty of Medicine, Chulalongkorn University, Bangkok 10330, Thailand; Excellence Center in Diabetes, Hormone, and Metabolism, King Chulalongkorn Memorial Hospital, Thai Red Cross Society, Bangkok 10330, Thailand

**Keywords:** type 1 daysiabetes, automated insulin delivery, hemodialysis, end stage kidney disease, intradialytic hypoglycemia, glycemic variability

## Abstract

Managing patients with type 1 diabetes (T1D) with end stage kidney disease (ESKD) on hemodialysis presents unique challenges due to altered insulin kinetics and rapid glucose fluxes. We report the case of a 48-year-old female with long-standing T1D and ESKD who suffered from severe glycemic variability and recurrent hypoglycemia despite intensive insulin management. The patient was transitioned to the Medtronic MiniMed™ 780G automated insulin delivery (AID) system. Although glycemic control generally improved, she experienced frequent intradialytic hypoglycemia and hypotension. A specialized protocol was implemented to mitigate these risks: setting a temporary glucose target of 150 mg/dL (SI: 8.3 mmol/L) (reference range, 70-180 mg/dL [SI: 3.9-10 mmol/L]) two hours prior to dialysis, suspending the pump upon dialysis initiation, and resuming delivery only when sensor glucose exceeded 200 mg/dL (SI: 11.1 mmol/L). Following this adjustment, intradialytic adverse events were resolved, and the patient achieved a time in range of 69% with a glucose management indicator (GMI) of 7.0%. This case demonstrates that while standard AID algorithms are not calibrated for dialysis, they can be safely employed in ESKD patients through tailored modifications, specifically strategic pump suspension and proactive target elevation.

## Introduction

Automated insulin delivery (AID) systems represent a significant advancement in diabetes technology, offering improved time in range (TIR), reduced glycemic variability and improved quality of life. However, the application of standard AID algorithms in patients with end-stage kidney disease (ESKD) undergoing hemodialysis (HD) is complicated by the risk of rapid glucose fluxes and “pseudo-hypoglycemia”— phenomenon where measured glucose is artifactually lower than true systemic levels due to impaired microcirculation or in vitro glycolysis during dialysis sessions [1]. Currently, no commercial AID systems are approved for people with ESKD. While limited trials have explored closed-loop systems in patients with type 2 diabetes (T2D) on dialysis, there is a paucity of real-world data regarding patients with type 1 diabetes (T1D) on HD. Recent case series suggest that with conservative settings—such as higher glucose targets and extended active insulin time (AIT), AID can be safely implemented in this cohort to improve TIR [2, 3]. We report a case illustrating the specific parameter adjustments required to safely utilize AID in person living with T1D undergoing HD.

## Case presentation

A 48-year-old Thai female with a 38-year history of T1D presented for management of severe glycemic variability and recurrent, severe hypoglycemia. Her medical history was notable for proliferative diabetic retinopathy, Hashimoto thyroiditis, atrial fibrillation, dyslipidemia, and ESKD secondary to diabetic nephropathy, requiring HD three times weekly. She was an active candidate on the waiting list for simultaneous pancreas-kidney (SPK) transplantation. She previously used a basal-bolus regimen consisting of insulin degludec 5 units at 20:00 along with insulin aspart 4–8 units according to carbohydrate intake. Despite this regimen, her glucose levels were erratic with frequent hypo- and hyperglycemia. Her blood glucose values ranged from 58 to 472 mg/dL (SI: 3.2—26.2 mmol/L) and reported frequent nocturnal and morning hypoglycemia. Due to frequent hypoglycemia and impaired hypoglycemia awareness, continuous glucose monitoring (CGM, Medtronic Guardian™4) was initiated. This device is not specifically approved for use in patients on HD, and published data on CGM accuracy in the dialysis setting are limited; therefore, we interpreted sensor readings cautiously and corroborated trends with capillary glucose checks during dialysis as needed.

## Diagnostic assessment

The initial CGM report revealed a TIR of 61%, time-below-range (TBR) of 4% and significant nocturnal hypoglycemia. Over the subsequent years, her insulin regimen was adjusted multiple times, and she was instructed to consume a bedtime carbohydrate to prevent nocturnal hypoglycemia. Despite the intervention, nocturnal hypoglycemia improved but persisted, with TBR 3-7%. Later, she switched to insulin glargine-U100 administered twice daily (3 units at 8:00 and 20:00 on non-HD days, 2 units at 8:00 and 3 units at 20:00 on HD days). She utilized insulin aspart for correction with an insulin-to-carbohydrate ratio (ICR) of 1 unit of insulin per 3 grams of carbohydrate (1:3) in the morning and 1:2 in the evening, alongside an insulin sensitivity factor (ISF) correction of 1.5 units for glucose >350 mg/dL (SI: 19.4 mmol/L). Diabetes self-management education emphasizing hypoglycemia prevention was implemented. Despite all attempts, she still experienced frequent and nocturnal hypoglycemia. CGM data from January to February 2025 (88% sensor wear) showed a TIR of 45-52%, TBR of 3-6%, and a glucose management indicator (GMI) of 7.8%. Glycemic variability was high (average glucose 229 ± 99 mg/dL, SI:12.7 ± 5.5 mmol/L; coefficient of variation [CV] 41%), with 1-2 low glucose alerts daily.

## Treatment

**Figure 1:**
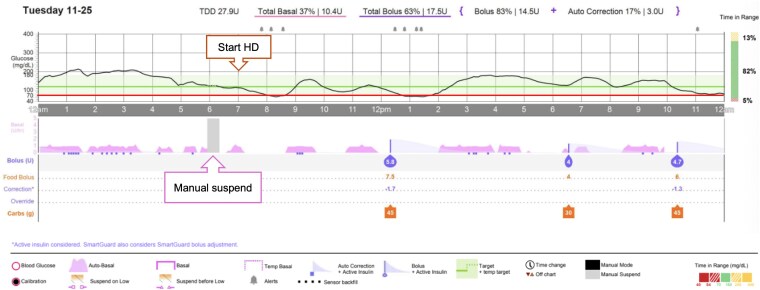
Glucose profile from AID during HD day before pre-dialysis protocol initiated.

Given the uncontrolled T1D, high glycemic variability, and recurrent hypoglycemia, therapy was escalated to an AID (Medtronic MiniMed™ 780G) in May 2025. The system was initially started in manual mode with insulin aspart. Initial setting based on weight included a basal rate of 0.4 units/h, ICR of 1:5 (8:00-18:00) and 1:7.5 (18:00-8:00), ISF of 90 mg/dL (SI: 5 mmol/L), and a target range of 100-150 mg/dL (SI: 5.6-8.3 mmol/L) with the “Suspend Before Low” feature enabled (threshold 90 mg/dL, SI: 5 mmol/L). Smartguard™ had not been activated due to multiple medical issues and patient's reluctance to technology. Four months after initiating AID, due to post-prandial hypoglycemia which resulted in TIR of 59-65%, TBR 1%, GMI 7.2-7.4%, the ICR was adjusted to 1:6 (8:00-18:00) and 1:7.5 (18:00-8:00). SmartGuard™ was also activated in this visit with a glucose target of 120 mg/dL (SI: 6.7 mmol/L) and an active insulin time of 3 hours. After activating SmartGuard™ with 92% usage for about 4 weeks, her TIR was 62%, TBR 1%, average blood glucose 237 ± 105 mg/dL (SI: 13.2 ± 5.8 mmol/L), GMI 7.4%, CV 41.5%, low blood glucose alert 2 times/day. However, at 3 months after starting SmartGuard™, she suffered from frequent intradialytic hypotension and hypoglycemia during HD sessions (scheduled at 07:00 on Tuesdays/Thursdays and 10:00 on Saturdays). AID reports indicated 96% SmartGuard™ usage, TIR 67–74%, TBR 1–2%, GMI 6.8–7.2%, and CV 37–38.4%, low glucose alerts 4-6 times daily. Detailed analysis revealed a distinct pattern of intradialytic hypoglycemia occurring concurrently with hypotension (Fig. 1), which the patient had attempted to manage by eating pre-dialysis and manually suspending the AID.

Thus, a specific intradialytic glycemic protocol was implemented:

1. Pre-dialysis: Set a Temporary Target of 150 mg/dL (SI: 8.3 mmol/L) for 2 hours prior to the HD session.
2. HD initiation: Suspend the AID pump upon initiation of HD.
3. Intradialytic management: Resume the AID pump—maintaining the Temporary Target—only once blood glucose from CGM exceeded 200 mg/dL (SI: 11.1 mmol/L).

## Outcome and follow-up

**Figure 2:**
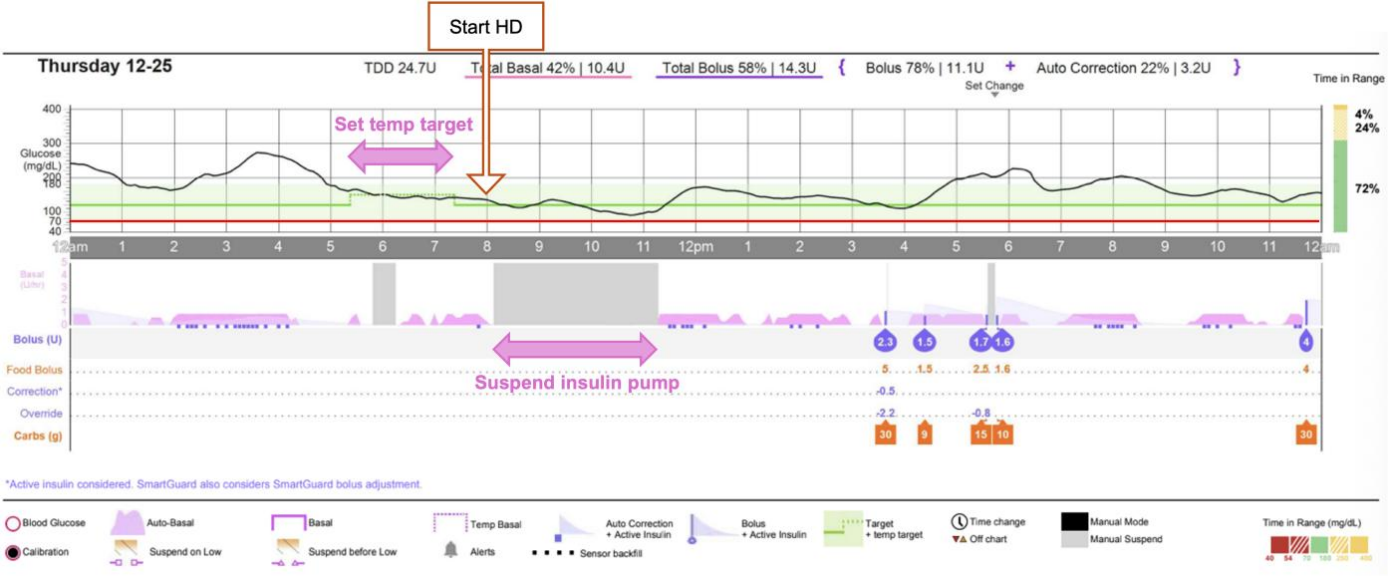
Glucose profile from AID during HD day after starting pre-dialysis glycemic management protocol.

One month after adjustment, intradialytic hypoglycemia improved, and hypotension resolved. AID reports following the new protocol (97% SmartGuard™ usage) demonstrated TBR<70 (Time below range <70 mg/dL (SI:3.9 mmol/L)) fell to 4%, TBR<54 (time below range <54 mg/dL (SI: 3 mmol/L)) to 2%, and %CV decreased to 37%, reflecting an overall reduction in hypoglycemia burden and less glycemic variability compared with baseline TBR<70 ranged 3–6% and TBR<54 ranged 1–2%, giving a combined low-glucose burden of approximately 5–8% with a %CV of 41%. Additional metrics were TIR 69%, GMI 7.0%, and frequency of low glucose alerts 7-8 times per day, but no severe intradialytic event, and reported improvement in patient well-being (Table 1 and Figs. 1 and  2).

**Table 1:** Comparing AID results between baseline, 4 weeks after starting AID with smartguard™, and 1 month after pre-HD protocol for AID

Parameter	Time point		
	Baseline (January-February2025)	AID report (4 weeks after SmartGuard™ initiated, September 2025)	AID report (SmartGuard™ initiated 1 month after pre-HD protocol initiated, January 2026)
Average glucose	229 ± 99 mg/dL (SI: 12.7 ± 5.5 mmol/L.)	237 ± 105 mg/dL (SI: 13.2 ± 5.8 mmol/L)	208 ± 105 mg/dL (SI: 11.5 ± 5.8 mmol/L)
TIR	45–52%	62%	69%
TBR<70	3–6%	1%	4%
TBR<54	1–2%	0%	2%
TAR>180	42–52%	37%	27%
TAR>250	13–21%	15%	7%
GMI	7.8%	7.4%	7%
%CV	41	41.5	37.4

Abbreviations: %CV, %coefficient of variation; AID, automated insulin delivery; GMI, glucose management indicator; HD, hemodialysis; TAR>180, time above range >180 mg/dL (SI: 10 mmol/L); TAR>250, time above range >250 mg/dL (SI: 13.9 mmol/L); TBR<54, time below range <54 mg/dL (SI: 3 mmol/L); TBR<70, time below range <70 mg/dL (SI:3.9 mmol/L); TIR, time in range.

## Discussion

We present a case that illustrates practical parameter adjustments required to safely deploy AID in a patient with T1D receiving maintenance HD. Patients with ESKD represent a uniquely high-risk group for hypoglycemia and marked glycemic variability due to altered insulin clearance, reduced gluconeogenic capacity, and dialysis-related shifts in glucose and fluid balance. Current AID algorithms were developed and validated in populations without advanced renal failure and therefore do not specifically address the physiologic perturbations encountered in ESKD. Published data with AID in ESKD is limited, with higher-quality data regarding AID available for patients with concurrent T2D and ESKD. In a randomized crossover trial comparing AID with standard insulin therapy in adults with T2D requiring HD, the AID group demonstrated a greater TIR and a reduced TBR <70 mg/dL [SI: 3.9 mmol/L] [4]. Available reports of patients with T1D with ESKD suggest that AID can improve TIR and reduce glycemic variability, and that systems are generally well tolerated when introduced within a multidisciplinary care framework [2, 3]. One case series proposed a clinical approach consisting of conservative treatment targets and AIT settings, and avoidance of bolus dosing to mitigate hypoglycemia [2]. However, detailed device settings and implementation strategies are rarely reported; to our knowledge only a small number of cases using AID (eg, Medtronic 780G) have been described, with scant information on the precise configuration and monitoring approaches employed [5]. Intradialytic hypotension and hypoglycemia are common among people with diabetes receiving HD and often occur together. Hypotension during hypoglycemia reflects autonomic dysfunction; hypoglycemia can impair the sympathetic response to hypotension, reducing vasoconstriction and heart rate compensation. Recurrent episodes of either condition, particularly hypoglycemia, are independently associated with increased mortality in patients on maintenance HD [6, 7].

The present case offers a pragmatic strategy to mitigate hypoglycemia and attenuate glucose excursions in this high-risk population by tailoring AID parameters to the dialysis schedule. Key components include proactive target adjustments around dialysis sessions, close glucose surveillance, and ongoing collaboration among endocrinology, nephrology, diabetes education, and nursing teams.

Although suspending insulin poses a risk of diabetic ketoacidosis (DKA), in our case, we minimized the duration of insulin pump suspension to only during HD, closely monitored CGM readings, and resumed insulin once glucose exceeded 200 mg/dL (SI: 11.1 mmol/L). The insulin suspension lasted 2-3 hours and did not extend beyond the dialysis session. No adverse events occurred with this strategy.

Despite the encouraging outcome in this single patient, AID settings must be individualized, and clinicians should apply caution given the lack of robust evidence. Prospective studies and registry data are needed to define optimal AID programming, safety profiles, and clinical outcomes in patients with T1D and ESKD.

## Learning points

- AID adaptation is mandatory: Standard AID algorithms are not calibrated for dialysis-induced hemodynamic shifts; manual modification is required to prevent intradialytic hypoglycemia.
- Effective intradialytic protocol: Hypoglycemia and hypotension were resolved by setting a temporary target of 150 mg/dL (SI: 8.3 mmol/L) at 2 hours pre-HD and suspending the pump during dialysis until glucose exceeded 200 mg/dL (SI: 11.1 mmol/L).
- Prioritize safety: In ESKD, avoiding hypoglycemia takes precedence over tight euglycemia. Conservative settings (higher targets, extended AIT) are necessary to buffer against rapid glucose fluxes.
- Clinical feasibility: AID (eg, Medtronic 780G) can safely improve TIR in HD patients when settings are highly individualized and closely monitored.

## Contributors

All authors made individual contributions to the authorship. R.A. was involved in patient follow-up, acquisition of clinical data, and interpretation of clinical findings. J.J. was involved in retrieving AID data and coordinating with the patient for follow-up and data collection. N.L. was involved in the diagnosis, management, and follow-up of the patient. All authors reviewed and approved the final draft.

## Data Availability

Some of the datasets generated during and/or analyzed during the current study are not publicly available but are available from the corresponding author on reasonable request.
